# Ultrasound‐Assisted DES Extraction of Black Rice Anthocyanins: Optimization, Characterization, and Bioactivity

**DOI:** 10.1111/1750-3841.71120

**Published:** 2026-05-08

**Authors:** Cuiqin li, Yabin Shi, Ziming Cao, Fengliang Liu

**Affiliations:** ^1^ College of Chemistry and Pharmaceutical Engineering Cangzhou Jiaotong University Cangzhou China; ^2^ School of Chemistry and Chemical Engineering Central South University Changsha China

**Keywords:** anthocyanins, antioxidant activity, black rice, deep eutectic solvents, hypoglycemic activity, response surface methodology, ultrasound‐assisted extraction

## Abstract

Black rice is a rich source of anthocyanins, which exhibit strong antioxidant properties and potential hypoglycemic effects. However, the application of black rice and related bioactive compounds in the food and functional food industries is limited by low extraction yields. Therefore, to promote the potential applications of black rice as a value‐added health product, the extraction, characterization, and evaluation of the beneficial effects of anthocyanins from black rice were examined in the present study. A green and efficient ultrasound‐assisted deep eutectic solvent extraction (UDE) method for extracting anthocyanins from black rice was established. Under the optimum conditions (choline chloride–lactic acid as DES with a mole ratio of 1:2.06, solid‐to‐liquid ratio of 1:19.8 g/mL, water content of 39.8%, and ultrasonic temperature of 50.8°C), a maximum yield (37.44 ± 0.04 mg/g) of anthocyanin extracted from black rice by UDE was obtained, which was significantly higher than ultrasound‐assisted ethanol extraction (UEE). LC‒MS analysis confirmed cyanidin‐3‐glucoside as the predominant component. In addition, compared with UEE, UDE exhibited much higher antioxidant effects as well as inhibitory effects against α‐glucosidase and α‐amylase. Collectively, the findings suggest that the UDE method can be applied as an efficient technique for the preparation of anthocyanins from black rice.

**Practical Applications:**

The optimized DES‐UAE extraction protocol directly addresses the bottleneck of low extraction yields associated with black rice anthocyanins, offering a highly efficient and safer alternative to conventional ethanol‐based methods by circumventing flammability and regulatory constraints. By avoiding the chemical degradation typically caused by traditional organic solvents, this green approach yields C3G‐enriched extracts with their inherent chemical integrity and bioactivity well‐maintained. Specifically, leveraging their demonstrated inhibitory effects against α‐glucosidase and α‐amylase, these extracts can be directly formulated into targeted dietary supplements for blood glucose management. Additionally, their robust antioxidant profile makes them suitable as clean‐label natural colorants for functional foods. Ultimately, this method provides a practical processing route for converting underutilized black rice into value‐added health products, supporting green manufacturing practices at the laboratory and pilot scales.

**Abbreviations**
BRA: black rice anthocyanin
ChCl: choline chloride
DES: deep eutectic solvents
DNS: dinitrosalicylic acid
DPPH: 1,1‐diphenyl‐2‐picrylhydrazyl
DTT: dithiothreitol
EDTA: ethylenediaminetetraacetic acid
ESI: electrospray ionization
HBA: hydrogen bond acceptors
HBD: hydrogen bond donors
HPLC: high‐performance liquid chromatography
MS: mass spectrometry
PBS: phosphate buffer solution
*p*‐NPG: *para*‐nitrophenyl‐α‐d‐glucopyranoside
PR: pyrogallol
RSM: response surface methodology
Tris: trihydroxy‐methyl aminomethane
UDE: ultrasound‐assisted deep eutectic solvent extraction
UEE: ultrasound‐assisted ethanol extraction.
VC: vitamin C

## 1 Introduction

Black rice is a distinctive cereal grain characterized by its dark purple to black pericarp, which is rich in anthocyanins. Owing to its nutritional and medicinal value, black rice has long been regarded in Asia as a functional food, often referred to as “medicinal rice” or “longevity rice” (Murthy et al. 2024). In recent years, increasing attention has been given to black rice anthocyanins (BRAs) due to their potent biological activities and potential applications in functional food and nutraceuticals (Guo et al. 2025).

Anthocyanins are water‐soluble flavonoids with a C6‐C3‐C6 biphenyl‐2‐pyran cation skeleton (S. Chen et al. 2024; X. Q. Chen et al. 2012), and more than 700 anthocyanin structures have been identified to date. In black rice, cyanidin‐3‐glucoside (C3G) is the predominant anthocyanin, accounting for approximately 90%–95% of the total anthocyanin content, followed by minor components such as peonidin‐3‐glucoside and various phenolic acids (Cai et al. 2023; Ghasemzadeh et al. 2018; Pattananandecha et al. 2021). The strong antioxidant capacity of anthocyanins is mainly attributed to their *o*‐dihydroxy phenolic structure (Salehi et al. 2020; Smeriglio et al. 2016; Yang et al. 2022), which enables efficient scavenging of reactive oxygen species. Numerous studies have demonstrated that BRAs exhibit a wide range of biological activities, including antioxidant (Ao and Kim 2020; C. Wang et al. 2022), anti‐inflammatory, cardioprotective (Curtis et al. 2019), neuroprotective (Di Giacomo et al. 2012), anticancer (AlMadalli et al. 2024; Silva et al. 2019), and glucose‐lowering effects (Corrêa et al. 2019).

Efficient extraction of anthocyanins is a critical prerequisite for their utilization. Conventional extraction methods primarily rely on organic solvents such as ethanol or methanol, often assisted by ultrasound (Cai et al. 2023; Zevallos et al. 2020), or microwave irradiation (Mondal et al. 2024; Zou et al. 2012). Although these methods are effective, they suffer from several drawbacks, including high solvent consumption, potential toxicity (Xie et al. 2019), environmental pollution, and solvent residue issues (Allison and Simmons 2018). As a result, the development of green, safe, and efficient extraction technologies has become an important research focus. Recently, increasing concern for ecological sustainability and the green economy has driven interest in deep eutectic solvents (DESs) as novel solvents for extracting phenolic compounds from plants (Meenu et al. 2023).

DES, first proposed by Abbott et al. (2003), has emerged as a new class of green solvents composed of HBA and HBD (Li et al. 2024). DESs are characterized by negligible volatility, easy preparation, tunable physicochemical properties, and excellent biodegradability (Petreska Stanoeva et al. 2024). Importantly, DESs can form strong hydrogen‐bonding interactions with phenolic compounds, facilitating the disruption of plant cell walls and enhancing the solubility and mass transfer of target compounds (Hikmawanti et al. 2021; Ozturk et al. 2018). Previous studies have demonstrated that DES‐based extraction is a promising alternative to conventional organic solvents for the recovery of bioactive compounds, including polyphenols and anthocyanins (Sereshti et al. 2022).

Ultrasound‐assisted extraction (UAE) further enhances extraction efficiency by inducing cavitation effects, which improve solvent penetration and accelerate the release of intracellular compounds. The combination of UAE with DESs has shown significant potential for improving extraction yield while reducing energy consumption and environmental impact (Arruda et al. 2021).

However, a critical challenge associated with DES‐based extraction is that the inherent high viscosity of certain DESs can act as a severe mass transfer barrier, potentially counteracting their solvation benefits. Therefore, it is hypothesized that coupling DES with ultrasound generates a pronounced physicochemical synergy, whereby intense acoustic cavitation is crucial for overcoming the viscous resistance of the DES matrix to facilitate efficient anthocyanin release. However, realizing this synergistic potential is highly dependent on the formulation of the DES itself. Specifically, the extraction efficiency is strongly dictated by multiple factors, such as solvent composition, molar ratio, and water content.

Therefore, systematic optimization of these parameters is essential to elucidate this synergistic mechanism (Rahman et al. 2025). Response surface methodology (RSM), particularly the Box‒Behnken design (BBD), is widely utilized to optimize extraction variables and evaluate their interactive effects while avoiding the need for an excessively large number of experiments (Shehzadi et al. 2026). However, the mechanistic optimization of a DES‐ultrasound combined extraction (UDE) system for BRAs and its subsequent impact on the functional integrity of the extracts have not yet been reported. Therefore, the present study was designed to first optimize the UDE parameters via BBD–RSM, elucidating how the interactions between DES physicochemical properties and ultrasonic cavitation govern the extraction yield. Subsequently, the extracted anthocyanins were characterized via LC‒MS to determine whether the optimized UDE microenvironment selectively enriches target bioactive compounds. Furthermore, the in vitro antioxidant and enzyme inhibitory activities were evaluated, thereby establishing a direct link between the tailored UDE process and the functional properties of BRAs. Ultimately, this mechanism‐driven approach provides a robust scientific basis for the green extraction of high‐value cereal‐derived ingredients.

## 2 Materials and Methods

### 2.1 Materials and Reagents

Black rice was obtained from Baoshan, Yunnan Province, China. Cyanidin‐3‐O‐glucoside (≥ 98%) was purchased from Shanghai Shifeng Biotechnology Co. Ltd. (Shanghai, China). AB‐8 macroporous resin was purchased from Shanghai Fushen Biotechnology Co. Ltd. (Shanghai, China). 1,1‐Diphenyl‐2‐picrylhydrazyl (DPPH) (≥ 97%), ascorbic acid (≥ 99%), pyrogallol (≥ 99%), dithiothreitol (DTT; ≥ 98%), Tris (≥ 99%), and other analytical‐grade reagents were supplied by Sinopharm Chemical Reagent Co. Ltd. (Beijing, China). α‐Amylase (≥ 1 U/mg) and α‐glucosidase (≥ 10 U/mg) were purchased from Macklin Biochemical Co. Ltd. (Shanghai, China). HPLC‐grade solvents were obtained from Merck (Darmstadt, Germany).

### 2.2 Sample Preparation

#### 2.2.1 Defatting of Black Rice Powder

Black rice grains (Baoshan, Yunnan Province, China) were ground into a fine powder and passed through a 60‐mesh sieve. The powder was defatted by mixing with petroleum ether (boiling range 60°C–90°C) at a solid‐to‐solvent ratio of 1:10 (w/v) and stirred or shaken at room temperature for 4 h. The residue was collected by vacuum filtration (SHZ‐D, Shanghai Lichen Bangxi Instrument Technology Co. Ltd., Shanghai, China; filter paper specifications: qualitative, medium speed, 9 cm), and the defatting procedure was repeated until the filtrate became colorless. The defatted powder was dried in a vacuum oven (Shanghai Lichen Bangxi Instrument Technology Co. Ltd.) at 40°C to constant weight, regrounded, sieved, and kept at −20°C until use.

#### 2.2.2 UAE of Anthocyanins

Defatted black rice powder (1.0 g) was mixed with the extraction solvent (DES or 70% ethanol) according to the designed solid‐to‐liquid ratio. The mixture was subjected to UAE using an ultrasonic bath (Guangzhou Kemeng Cleaning Technology Co. Ltd.) under specified temperature, power, and time conditions. After extraction, the suspension was cooled to room temperature and centrifuged (4120 × *g*, 10 min, 4°C) (Liu et al. 2012; Y. Wang et al. 2020). The supernatant was collected, concentrated (Shanghai Lichen Bangxi Instrument Technology Co. Ltd.) under reduced pressure below 40°C, and freeze‐dried (Shanghai Lichen Bangxi Instrument Technology Co. Ltd.) to obtain crude anthocyanin extracts.

#### 2.2.3 Determination of Anthocyanin Content

The total anthocyanin content was determined using the pH differential method. Freeze‐dried extracts were dissolved and diluted with a pH 1.0 potassium chloride buffer and a pH 4.5 sodium acetate buffer. After equilibration in the dark at 30°C for 30 min, absorbance was measured at 520 and 700 nm. The anthocyanin content was calculated and expressed as C3G equivalents (mg/g dry weight) (Postolache et al. 2023; Qin et al. 2024).

$$\begin{array}{ccc} & & Y\left(\mathrm{mg}/g\right)=\\ & & \frac{\left(A_{520\;\mathrm{nm},\mathrm{pH}\;1.0}-A_{700\;\mathrm{nm},\mathrm{pH}\;1.0}\right)-\left(A_{520\;\mathrm{nm},\mathrm{pH}\;4.5}-A_{700\;\mathrm{nm},\mathrm{pH}\;4.5}\right)}{\varepsilon \times L\times m}\\ & & \times \mathrm{MW}\times \mathrm{DF}\times 1000 \end{array}$$

In the equation, *A* denotes the absorbance. MW represents the molecular weight of the predominant anthocyanin in black rice, C3G (449.2 g/mol). DF is the dilution factor, and *ε* is the mole extinction coefficient of C3G at pH 1.0 and 520 nm (26,900 L/mol cm). *L* denotes the optical path length (1 cm), and *m* represents the mass of the lyophilized sample used for analysis (g).

### 2.3 Preparation and Screening of DES

Choline chloride (Sinopharm Chemical Reagent Co. Ltd.) was used as the hydrogen bond acceptor (HBA), while lactic acid, propylene, citric acid, glucose, sucrose, glycerol, and urea served as hydrogen bond donors (HBDs). DESs were prepared by heating and stirring the components at specified mole ratios until a homogeneous transparent liquid was obtained. The prepared DESs were cooled to room temperature and stored before use. Screening experiments were conducted by extracting 1.0 g of defatted black rice powder with different DESs (containing 30% water) or 70% ethanol at a solid‐to‐liquid ratio of 1:15 (g/mL), ultrasonic temperature of 50°C, power of 200 W, and extraction time of 40 min (Islamčević Razboršek et al. 2020; Shu et al. 2025). After thoroughly mixing these components, the mixture was transferred to stoppered conical flasks. Subsequently, these flasks were incubated in a thermostatic water bath at 100°C for sucrose and glucose and at 80°C for the other HBDs. The mixture was stirred continuously for different durations: 2 h for organic acids and polyols, 3 h for sugars, and 1 h for amines until it formed a clear, homogeneous liquid. The mixture was then cooled to room temperature, sealed, and stored for future use (Table 1). DES with the highest anthocyanin yield was selected for further optimization.

**TABLE 1:** Composition of deep eutectic solvents.

DES	Hydrogen bond acceptor	Hydrogen bond donor	Mole ratio
DES‐1	ChCl	Lactic acid	1:02
DES‐2	—	1,2‐Propylene glycol	1:03
DES‐3	—	Citric acid	1:01
DES‐4	—	Glucose	2:01
DES‐5	—	Sucrose	2:01
DES‐6	—	Glycerol	1:02
DES‐7	—	Urea	1:02

### 2.4 Single‐Factor Experiments and RSM

Single‐factor experiments were performed to evaluate the effects of the DES mole ratio, water content, solid‐to‐liquid ratio, ultrasonic temperature, power, and extraction time on anthocyanin yield. The ultrasonic power, temperature, and extraction time were set at 200 W, 50°C, and 40 min (during the heating process, we placed a thermometer at the bottom of the vessel to monitor the temperature in real time), respectively, with a solid‐to‐liquid ratio of 1:15 (g/mL). The mole ratio of HBA to HBD in the DES was fixed at 1:2. Using a single‐factor experimental design, the effects of extraction solvent (70% ethanol and the selected DES) on anthocyanin yield from black rice were first evaluated, and these two solvents were subsequently used for further experiments. The influences of key extraction parameters on anthocyanin yield were then investigated, including the DES mole ratio (3:1, 2:1, 1:1, 1:2, and 1:3), water content of DES (20%–60%), solid‐to‐liquid ratio (1:10–1:30 g/mL), ultrasonic temperature (30°C–70°C), extraction time (20–70 min), and ultrasonic power (100–300 W).

Based on these results, a BBD was applied using Design‐Expert 13 software to optimize four key variables: mole ratio (*A*), solid‒liquid ratio (*B*), water content (*C*), and ultrasonic temperature (*D*). The second‐order polynomial equation was determined for the response variable as follows:

$$Y=\beta_{0}+\sum_{i=1}^{k}\beta_{i}X_{i}+\sum_{i=1}^{k}\beta_{ii}X_{i}^{2}+\sum_{i=1}^{k}\sum_{j=i+1}^{k-1}\beta_{ij}X_{i}X_{j}$$

where *Y* is the response variable and *β*_0_ *, β_i_, β_ii_*, and *β_ij_* represent the regression coefficients of intercept, linear, quadratic and interaction, respectively. *X_i_* and *X_j_* are the independent variables. *k* is a variable number (*k* = 4).

### 2.5 Purification of BRA

The required amount of AB‐8 macroporous adsorbent resin (Shanghai Fushen Biotechnology Co. Ltd.) was accurately weighed. It was soaked in anhydrous ethanol for 24 h. Afterward, the resin was thoroughly rinsed with ultrapure water until the pH of the effluent matched that of the ultrapure water and no ethanol odor was detected. The activated AB‐8 resin (Xiao et al. 2013) was loaded into a glass chromatography column (2.5 cm × 40 cm) using the wet method. The sample solution was introduced into the column at a slow flow rate of 60 mL/h, with a loading volume of 50 g. Once the sample solution had fully entered the bed, the column was eluted with 200 mL of ultrapure water at an appropriate flow rate. After the ultrapure water had been completely eluted, a 60% aqueous ethanol solution was used to elute the target anthocyanin compound.

The elution was performed at a flow rate of 75 mL/h, and a volume of 50 mL was collected. The eluate was collected, concentrated, subjected to ethanol removal, and freeze‐dried to obtain the purified anthocyanin. The anthocyanin content before and after purification was assessed using the pH differential method, with three parallel experiments performed.

The AB‐8 macroporous adsorption resin was accurately weighed and soaked in anhydrous ethanol for 24 h for activation. The resin was then thoroughly rinsed with ultrapure water until the effluent reached a neutral pH and no ethanol odor was detected. The activated resin was packed into a glass chromatography column using the wet‐packing method, as described previously (Xiao et al. 2013).

The anthocyanin extract was loaded onto the column at a flow rate of 60 mL/h, with a loading amount of 50 g. After complete adsorption, the column was washed with 200 mL of ultrapure water to remove impurities. Subsequently, the target anthocyanins were eluted with 60% (v/v) aqueous ethanol at a flow rate of 75 mL/h, and a volume of 50 mL was collected. The eluate was concentrated under reduced pressure to remove ethanol and then freeze‐dried to obtain purified BRAs.

The anthocyanin content before and after purification was determined using the pH differential method. All measurements were performed in triplicate.

The purity of the anthocyanins was calculated according to the following equation:

$$\mathrm{Purity}\;\mathrm{of}\;\mathrm{anthocyanins}\left(\%\right)=\frac{C\times V}{M}\times 100\%$$

where *C* is the concentration of purified anthocyanins (mg/mL), *V* is the volume of the purified anthocyanin solution (mL), and *M* is the mass of the purified lyophilized powder (mg).

### 2.6 LC‒MS Analysis

LC–ESI–MS analysis was performed using an Agilent 1100 liquid chromatography system equipped with a ZORBAX SB‐C18 column (100 mm × 3.0 mm, 1.8 µm; Agilent, Beijing, China), following a previously reported method with minor modifications (Y. Wang et al. 2020). The system was coupled with a diode array detector (DAD) before the mass spectrometer to monitor chromatographic separation. The column temperature was maintained at 40°C, and the injection volume was 20 µL. The mobile phase consisted of 0.1% (v/v) formic acid in water (*A*) and acetonitrile (*B*), delivered at a flow rate of 0.4 mL/min using the following gradient program: 0–5 min, 5% B; 5–10 min, 5%–20% B; 10–20 min, 20%–40% B; 20–30 min, 40%–80% B; 30–35 min, 80% B; 35.1–40 min, 5% B.

Mass spectrometric detection was carried out using electrospray ionization in positive ion mode ([M+H]^+^). Nitrogen was used as the collision gas at a flow rate of 10 mL/min. The spray voltage was set at 4.5 kV, the capillary voltage at 10 V, and the capillary temperature at 250°C. Full‐scan mass spectra were acquired over a *m*/*z* range of 50–2000.

### 2.7 Antioxidant Activity Assays

Antioxidant activity was evaluated using total reducing power, DPPH (Sinopharm Chemical Reagent Co. Ltd.) radical scavenging, and superoxide anion scavenging assays. In vitro hypoglycemic activity was assessed by measuring α‐amylase (Macklin Biochemical Co. Ltd.) and α‐glucosidase (Macklin Biochemical Co. Ltd.) inhibitory activities. All experiments were performed in triplicate.

#### 2.7.1 Reducing Power Assay

Purified DES‐extracted anthocyanins, purified 70% ethanol extracts, and VC were prepared at concentrations of 0.1, 0.2, 0.4, 0.6, and 0.8 mg/mL using phosphate buffer (0.2 mol/L, pH 7.4). An aliquot of 1.0 mL sample solution was mixed with 1.0 mL of 1% (w/v) potassium ferricyanide and 1.0 mL of phosphate buffer (pH 6.6), followed by incubation at 50°C for 20 min. Subsequently, 1.0 mL of 10% (w/v) trichloroacetic acid was added, and the mixture was centrifuged.

An aliquot of 0.3 mL supernatant was mixed with 0.5 mL ultrapure water and 0.5 mL of 0.1% (w/v) ferric chloride solution. After standing for 2 min, absorbance was measured at 700 nm (*A*_S_). Ultrapure water was used as the blank control (*A*_0_). Reducing power was expressed as:

$$\mathrm{Total}\;\mathrm{reduction}\;\mathrm{potential}=A_{S}-A_{0}$$

#### 2.7.2 DPPH Radical Scavenging Activity

Purified anthocyanin samples, VC, and purified 70% ethanol extracts were prepared at concentrations of 0.1–0.8 mg/mL. Each sample solution was mixed with DPPH solution and incubated at room temperature in the dark for 30 min. Absorbance was measured at 517 nm.

The DPPH radical scavenging activity was calculated as follows:

$$\mathrm{DHHP}\;\mathrm{radical}\;\mathrm{scavenging}\;\mathrm{rate}\left(\%\right)=\left(1-\frac{A_{\mathrm{AS}}-A_{\mathrm{ASB}}}{A_{\mathrm{AC}}}\right)\times 100\%$$

where *A*_AC_ is the absorbance of the DPPH solution without the sample, *A*_AS_ is the absorbance of the reaction mixture containing both DPPH and the sample, and *A*_ASB_ is the absorbance of the sample without DPPH (Table 2).

**TABLE 2:** Experimental design for the assay for scavenging DPPH.

	*A*_AC_	*A*_ASB_	*A*_ASB_
DPPH (mL)	2.0	2.0	0
Distilled water (mL)	2.0	0	2.0
Sample solution (mL)	0	2.0	2.0

*Note: A*_AC_, sample free. *A*_ASB_, with DPPH and sample. *A*_ASB_, DPPH free.

#### 2.7.3 Superoxide Anion Scavenging Activity

Superoxide anion scavenging activity was determined using the pyrocatechol autoxidation method (Y. Wang et al. 2020). Tris‐HCl buffer (50 mmol/L, pH 8.5), EDTA solution (1 mmol/L), pyrocatechol solution (0.4 mmol/L), and DTT solution (100 mmol/L) were prepared using ultrapure water. Purified anthocyanin samples, VC, and purified 70% ethanol extracts were prepared at concentrations of 10, 25, 50, and 100 µg/mL (Table 3).

**TABLE 3:** Experimental design for the scavenging assay $O_{2}^{-}.$

	*A*_sample_	*A*_sample‐blank_	*A*_control_	*A*_blank_
Sample solution (mL)	0.4	0.4	0	0
PR (mL)	1.0	0	1.0	0
Distilled water (mL)	0	1.0	0.4	1.4

*Note: A*_sample_, with sample and PR. *A*_sample‐blank_, PR free, with sample. *A*_control_, sample free, with PR. *A*_blank_, sample free, PR free. Tris‐HCl and EDTA solutions were added at constant volumes of 1.0 mL each in all treatments.

The reaction mixture was incubated at 30°C for 10 min, and the reaction was terminated by adding 30 µL of DTT solution. The absorbance was measured at 320 nm. The superoxide anion scavenging rate was calculated as follows:

$$\begin{array}{ccc} & & \mathrm{Superoxide}\;\mathrm{anion}\;\mathrm{clearance}\;\mathrm{rate}\left(\%\right)=\\ & & \left(1-\frac{A_{\mathrm{sample}}-A_{\mathrm{sample}-\mathrm{blank}}}{A_{\mathrm{control}}-A_{\mathrm{blank}}}\right)\times 100\% \end{array}$$

### 2.8 In Vitro Hypoglycemic Activity Assays

#### 2.8.1 α‐Amylase Inhibitory Activity

The inhibitory effects of the anthocyanin extracts prepared by different extraction methods against α‐amylase were assessed according to a previous procedure (Deng et al. 2024). Purified anthocyanin samples, VC, and purified 70% ethanol extracts were prepared at different concentrations in PBS buffer (0.1 mol/L, pH 6.8). An aliquot of 50 µL sample solution was mixed with 50 µL α‐amylase solution (1 U/mL) and incubated at 37°C for 10 min. Subsequently, 150 µL of 1% (w/v) soluble starch solution was added and incubated at 37°C for 5 min. The reaction was terminated by adding 250 µL of DNS reagent, followed by boiling for 5 min. After cooling to room temperature, absorbance was measured at 540 nm (Table 4) (Chen et al. 2023).

**TABLE 4:** Experimental design for the assay of α‐amylase inhibition.

	*A*_1_	*A*_2_	*A*_3_	*A*_4_
Sample solution (µL)	50	50	0	0
α‐Amylase (µL)	50	0	50	0
Starch (µL)	150	150	150	150
PBS (µL)	0	50	50	100
DNS (µL)	250	250	250	250

*Note: A*_1_, PBS free, with sample. *A*_2_, α‐amylase free, with sample. *A*_3_, sample free, with α‐amylase. *A*_4_, sample free, α‐amylase free.

The α‐amylase inhibition rate was calculated as follows:

$$\alpha \text{-Amylase}\;\text{inhibition}\;\text{rate}\left(\%\right)=\left(1-\frac{A_{1}-A_{2}}{A_{3}-A_{4}}\right)\times 100\%$$

#### 2.8.2 α‐Glucosidase Inhibitory Activity

According to a previous procedure (Deng et al. 2024), the inhibitory effects of anthocyanin extracts prepared by different extraction methods against α‐glucosidase were assessed. Purified anthocyanin samples, VC, and purified 70% ethanol extracts were prepared in PBS buffer (0.1 mol/L, pH 6.8). Sample solution (20 µL) was mixed with 20 µL α‐glucosidase solution (1 U/mL) and incubated at 37°C for 10 min. Subsequently, 40 µL of *p*‐nitrophenyl‐α‐d‐glucopyranoside (*p*‐NPG) solution was added and incubated at 37°C for 20 min. The reaction was terminated by adding 80 µL of Na_2_CO_3_ solution (1 mol/L). Absorbance was measured at 405 nm (Table 5).

**TABLE 5:** Experimental design for the assay of α‐glucosidase inhibition.

	*A*_1_	*A*_2_	*A*_3_	*A*_4_
Sample solution (µL)	20	20	0	0
α‐Glucosidase (µL)	20	0	20	0
*p*‐NPG(µL)	40	40	40	40
Na_2_CO_3_ (µL)	80	80	80	80
PBS (µL)	0	20	20	40

*Note: A*_1_, PBS free, with α‐glucosidase. *A*_2_, α‐glucosidase free, with sample. *A*_3_, sample free, with α‐glucosidase. *A*_4_, sample free, α‐glucosidase free.

The α‐glucosidase inhibition rate was calculated as follows:

$$\alpha \text{-Glucosidase}\;\text{inhibition}\;\text{rate}\left(\%\right)=\left(1-\frac{A_{1}-A_{2}}{A_{3}-A_{4}}\right)\times 100\%$$

### 2.9 Statistical Analysis

Statistical analysis was performed using SPSS 27 and Design‐Expert 13 software. The results are expressed as the mean ± standard deviation (*n* ≥ 3). Statistical analysis was performed by one‐way ANOVA or a two‐tailed Student's *t*‐test. The threshold for statistical significance was set at *p* < 0.05.

## 3 Results and Discussion

### 3.1 Screening of DES

#### 3.1.1 Selection of DES Preparation Reagents

**FIGURE 1:**
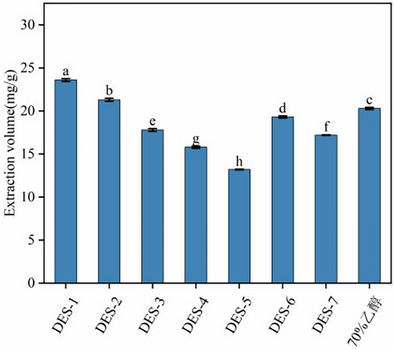
The impact of various DES solvents on the extraction yield of anthocyanins from black rice.

The physicochemical properties of DESs, including composition, acidity/alkalinity, viscosity, and polarity, play a critical role in determining the extraction efficiency of BR, which is closely related to the nature of HBD. As shown in Figure 1, significant differences in BRA yields were observed among the tested DES systems. The DES formulated with sucrose as the HBD exhibited the lowest BRA extraction yield (13.18 ± 0.05 mg/g), which was even lower than that obtained using conventional organic solvents.

In general, DESs containing acidic HBDs showed markedly higher extraction efficiencies than those with basic HBDs. Specifically, the extraction yields of acidic DES‐1 and DES‐3 were significantly higher than those of the basic DES‐7. Acidic HBDs increase the polarity of DESs, thereby enhancing their affinity for polar anthocyanins. Notably, the DES composed of choline chloride and lactic acid (DES‐1) achieved the highest BRA extraction yield (23.64 ± 0.17 mg/g).

Anthocyanins possess multiple hydroxyl groups and conjugated structures with both acidic and basic functional moieties, making them highly soluble in polar solvents, particularly under acidic conditions. The solubility and diffusion behavior of anthocyanins in DESs are influenced by several factors, among which solvent polarity plays a dominant role. DES‐1 exhibits strong polarity, which effectively enhances the overall polarity of the extraction system and promotes efficient mass transfer (Alrugaibah et al. 2021). This favorable solvent environment facilitates the stable dissolution of BRA, ultimately leading to improved extraction efficiency. Based on these results, DES‐1 was selected as the optimal extraction solvent for subsequent experiments.

### 3.2 Effects of Single‐Factor Variables on BRA Extraction

#### 3.2.1 Effect of DES Mole Ratio

**FIGURE 2:**
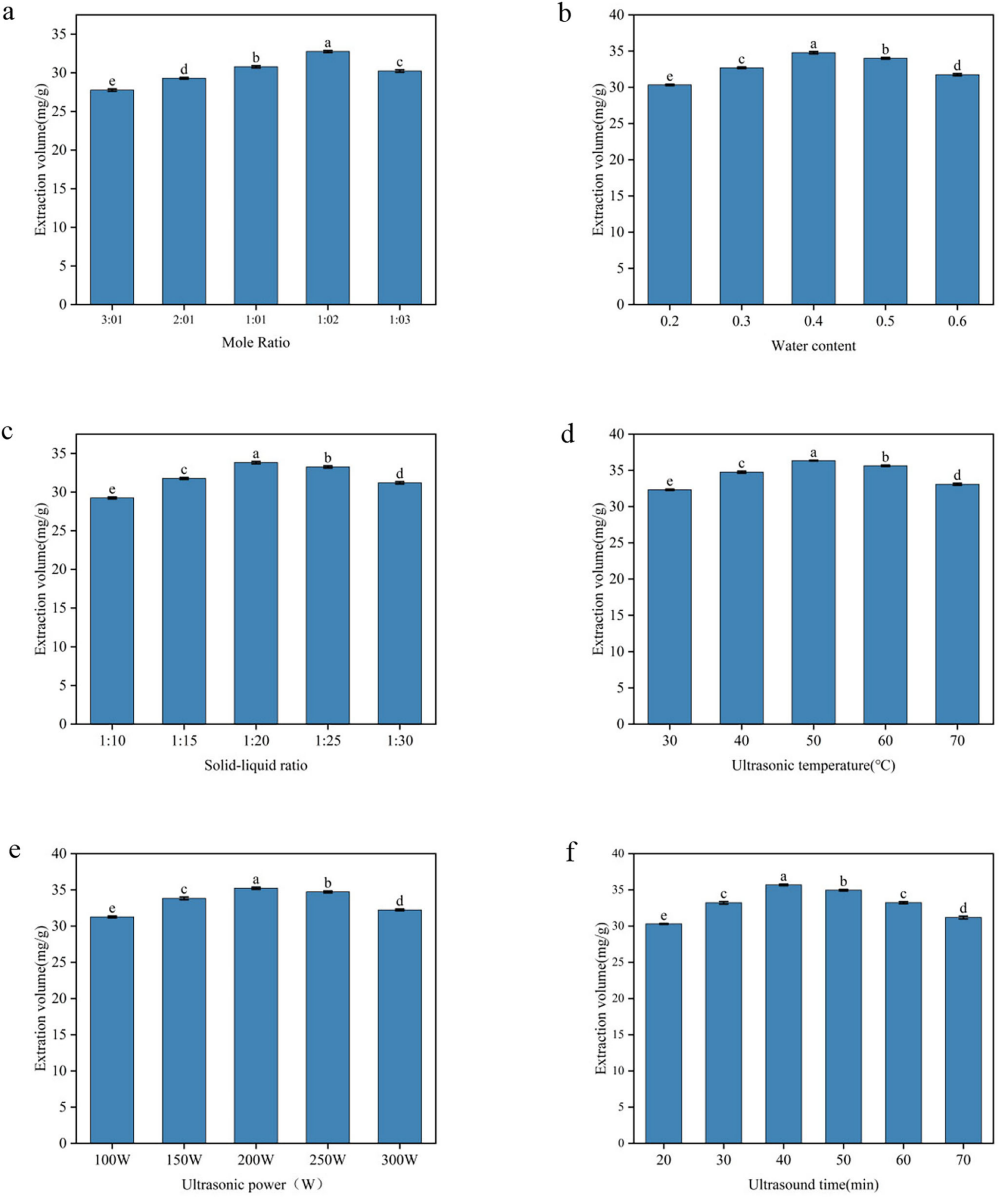
Effects of single‐factor parameters on ultrasound‐assisted deep eutectic solvent extraction of black rice anthocyanins (BRAs): (a) DES mole ratio, (b) water content, (c) solid‒liquid ratio, (d) ultrasonic temperature, (e) ultrasonic power, and (f) ultrasonication time. Different letters indicate significant differences (*p* < 0.05) as determined by one‐way ANOVA.

As shown in Figure 2a, the extraction yield of BRAs was significantly affected by the mole ratio of HBA to HBD (*p* < 0.05). The BRA yield initially increased with increasing HBD proportion and reached a maximum value of 32.76 ± 0.15 mg/g at a mole ratio of 1:2, after which a noticeable decline was observed.

The initial increase can be attributed to the optimal formation of hydrogen bond networks between HBA and HBD, which enhances solvent polarity and improves the solubilization of anthocyanins. However, excessive HBD content disrupts the balance of intermolecular interactions within the DES system, leading to increased viscosity and competitive hydrogen bonding, thereby reducing anthocyanin solubility and extraction efficiency. Based on these results, a mole ratio of 1:2 was selected for subsequent experiments.

#### 3.2.2 Effect of Water Content

The effect of the water mass fraction in DES on the BRA extraction yield is presented in Figure 2b. Water plays a dual role in DES‐based extraction. Moderate water addition reduces solvent viscosity and enhances mass transfer, facilitating anthocyanin diffusion. Consistently, the BRA extraction yield increased significantly when the water content in the DES was raised from 20% to 40%. However, the BRA extraction yield decreased significantly when the moisture content was further increased from 40% to 60%. The extraction efficiency of phenolic compounds was enhanced by adding an appropriate amount of water to the DES solution, which reduced the viscosity of the mixture (Meng et al., 2025. Therefore, based on the results obtained, the DES solution with a water content of 40% was ultimately selected for the subsequent single‐factor experiments.

#### 3.2.3 Effect of Solid‐to‐Liquid Ratio

As illustrated in Figure 2c, as the ratio increased from 1:10 to 1:20 g/mL, the yield of anthocyanins increased. However, when the sample‐to‐liquid ratio increases further, the yield decreases again.

Excessive solvent usage may lead to the co‐extraction of impurities, dilution of target compounds, and attenuation of ultrasonic energy transmission, ultimately reducing extraction efficiency (Yin et al. 2020). Therefore, the optimal sample‐to‐liquid ratio ranges from 1:10 to 1:30 g/mL to achieve the highest anthocyanin yield.

#### 3.2.4 Effect of Ultrasonic Temperature

As illustrated in Figure 2d, the BRA yield increased with temperature from 30 to 70°C and reached a maximum of 36.34 ± 0.05 mg/g at 50°C (*p* < 0.05). Further temperature increases led to a decline in extraction yield.

Moderate heating reduces DES viscosity and enhances molecular diffusion, thereby improving extraction efficiency. However, excessive temperatures may cause thermal degradation of anthocyanins and destabilization of solvent–solute interactions, resulting in reduced extraction efficiency (Meng et al., 2025. Therefore, 50°C was identified as the optimal extraction temperature.

#### 3.2.5 Effect of Ultrasonic Power

As shown in Figure 2e, ultrasonic power exerted a significant influence on BRA extraction (*p* < 0.05). Increasing the ultrasonic power from 100 to 200 W also increased the BRA extraction yield. The maximum BRA extraction yield was obtained at an ultrasonic power of 200 W; beyond this value, the BRA extraction yield began to decline. Generally, an increase in ultrasonic power may lead to higher hydrodynamic forces, which can significantly disrupt cell walls and enhance the extraction process. However, excessively high ultrasonic power may lead to an increase in the number of bubbles formed during the cavitation process, thereby reducing the energy efficiency transferred to the solvent (Linares and Rojas 2022).

#### 3.2.6 Effect of Ultrasonication Time

The effect of ultrasonication time on BRA extraction yield is presented in Figure 2f. The extraction yield increased significantly with time and reached a maximum of 35.69 ± 0.12 mg/g at 40 min (*p* < 0.05), after which a decline was observed.

Prolonged ultrasonication initially enhances mass transfer and accelerates the release of anthocyanins by disrupting cell structures. However, once the extraction equilibrium is reached, excessive ultrasonication may promote oxidative and thermal degradation of anthocyanins due to prolonged exposure to ultrasound, light, and oxygen, leading to reduced yields (Xue et al. 2020a, 2020b). Therefore, 40 min was selected as the optimal ultrasonication time.

### 3.3 RSM Optimization

#### 3.3.1 Experimental Design for Response Surface Analysis

Based on the results of single‐factor experiments and considering experimental cost as well as factor interactions, a four‐factor, three‐level BBD was employed to optimize the ultrasound‐assisted DES extraction of BRA. The selected independent variables were the DES mole ratio (*A*), solid‒liquid ratio (*B*), water content (*C*), and ultrasonic temperature (*D*), while the BRA extraction yield (mg/g) was used as the response variable. The experimental design matrix and corresponding results are summarized in factor levels and the results of Box‒Behnken experiments (details regarding this point are provided in Supporting Information).

#### 3.3.2 Regression Model and Analysis of Variance

The experimental data were analyzed using Design‐Expert 13 software, and a second‐order polynomial regression model was established to describe the relationship between the response and the independent variables. The regression equation for BRA extraction yield (*Y*) is expressed as:

$$\begin{array}{ccc} Y & = & -192.01442+16.01233A+6.26677B+3.34438C+3.32208D\\ & & +0.097000AB-0.083000AC+0.056250AD+0.005250BC\\ & & \;-\;0.015300BD+0.000825CD-4.24433A^{2}-0.148873B^{2}\\ & & -0.041706C^{2}-0.031181D^{2}. \end{array}$$

where *Y* represents the anthocyanin yield (mg/g) and *A*, *B*, *C*, and *D* are the coded independent variables for the mole ratio, solid‒liquid ratio, water content, and ultrasonic temperature, respectively. The regression coefficients of the model are represented by the numerical values preceding the variables.

As indicated in Table 6, the fitted model exhibited a high *F* value (668.94) and an extremely low *p* value (< 0.0001). Therefore, the model was statistically significant (Boateng et al. 2023). In contrast, the lack‐of‐fit test yielded an *F* value of 1.14 and a *p* value of 0.4885, indicating that the fitted model could accurately predict response values (Boateng et al. 2023; Bezerra et al. 2019). Furthermore, the low coefficient of variation (CV = 0.54%) indicated small fluctuations in response values, thereby confirming the high accuracy and reliability of the fitted model (Boateng et al. 2023; Bezerra et al. 2019). The low CV (0.54%) further demonstrated the model's excellent fit and predictive accuracy. Meanwhile, the coefficient of determination (*R* ^2^) and adjusted *R* ^2^ were 0.9930 and 0.9970, respectively, further confirming the model's reliability (Boateng et al. 2023; Bezerra et al. 2019). Under these extraction conditions, the anthocyanin yield from black rice using the UDE method was 37.74 mg/g (*n* = 3), which closely matched the predicted value of 37.44 mg/g. The relative standard deviation, which is below 10%, demonstrated the model's consistency and reliability. Overall, the regression model was valid.

**TABLE 6:** Analysis of variance results of the regression model.

Source	Sum of squares	df	Mean square	*F*	*p*	
Model	260.57	14	18.61	668.94	< 0.0001	significant
*A*—Mole ratio	2.62	1	2.62	94.26	< 0.0001	**
*B*—Solid‒liquid ratio	0.7252	1	0.7252	26.07	0.0002	*
*C*—Water content	0.168	1	0.168	6.04	0.0276	*
*D*—Ultrasonic temperature	2.27	1	2.27	81.61	< 0.0001	**
*AB*	0.9409	1	0.9409	33.82	< 0.0001	**
*AC*	2.76	1	2.76	99.04	< 0.0001	**
*AD*	1.27	1	1.27	45.49	< 0.0001	**
*BC*	0.2756	1	0.2756	9.91	0.0071	*
*BD*	2.34	1	2.34	84.14	< 0.0001	**
*CD*	0.0272	1	0.0272	0.9785	0.3394	
*A* ^2^	116.85	1	116.85	4199.76	< 0.0001	**
*B* ^2^	89.85	1	89.85	3229.38	< 0.0001	**
*C* ^2^	112.82	1	112.82	4055.08	< 0.0001	**
*D* ^2^	63.06	1	63.06	2266.63	< 0.0001	**
Residual	0.3895	14	0.0278			
Lack of fit	0.2884	10	0.0288	1.14	0.4885	not significant
Pure error	0.1011	4	0.0253			
Cor total	260.96	28				

*Note*: Predicted *R* ^2^ = 0.9930, CV% = 0.54. Statistical significance is indicated as *p < 0.05, **p < 0.01.

The simulation's coefficient of determination (*R* ^2^ = 0.9930) indicates a strong correlation between the model and experimental results. Residual analysis further confirmed the adequacy of the model. As illustrated in Figure 4, the residuals were normally distributed and closely aligned with the fitted line, indicating no apparent deviation from normality and validating the suitability of the regression model for predicting BRA extraction yield.

According to the ANOVA results (detailed data are available in Supporting Information), the linear terms *A*, *B*, *C*, and *D*, as well as the quadratic terms *A* ^2^, *B* ^2^, *C* ^2^, and *D* ^2^, had extremely significant effects on BRA extraction yield (*p* < 0.01). Based on the *F* values, the influence of the four factors on BRA extraction yield followed the order: DES mole ratio > ultrasonic temperature > solid‐to‐liquid ratio > water content.

#### 3.3.3 Interaction Effects and Response Surface Analysis

**FIGURE 3:**
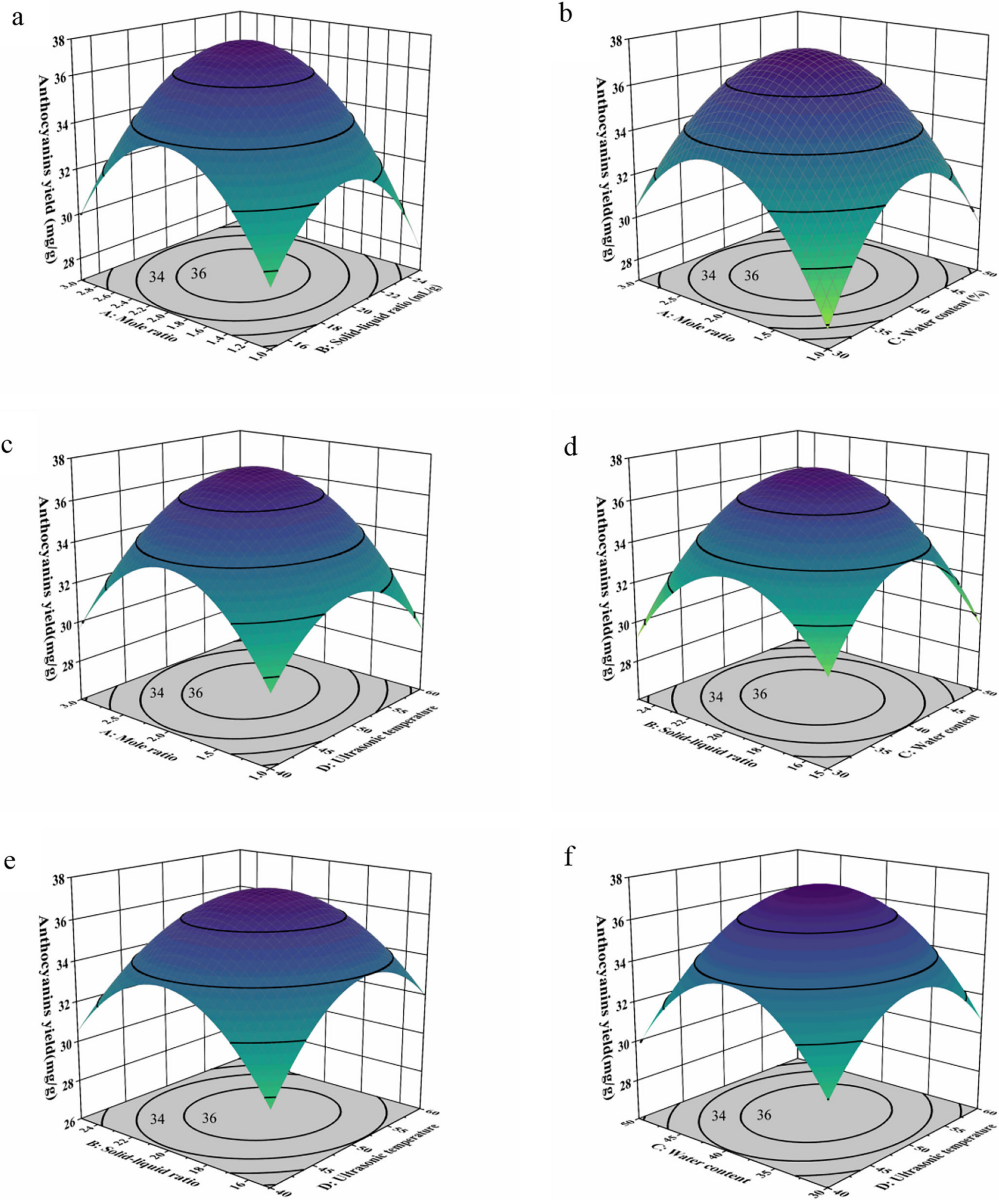
3D response surface plots for each interaction (a, mole ratio—solid‒liquid ratio; b, mole ratio—water content; c.: mole ratio—ultrasonic temperature; d, water content—Solid‒liquid ratio; e, water content—ultrasonic temperature; f, solid‒liquid ratio—Ultrasonic temperature).

Three‐dimensional response surfaces and corresponding contour plots (Figure 3) were generated to elucidate the intricate interactions among the DES–UAE variables and their mechanistic impact on BRA extraction yield. Notably, pronounced curvature and tight elliptical contours were observed for several key factor combinations—namely, DES mole ratio with solid‐to‐liquid ratio, DES mole ratio with water content, and DES mole ratio with ultrasonic temperature (*p* < 0.01)—which visually underscore a high degree of interdependence. These statistically significant interactions are not merely numerical phenomena but rather reflect a critical physicochemical synergy within the extraction microenvironment.

**FIGURE 4:**
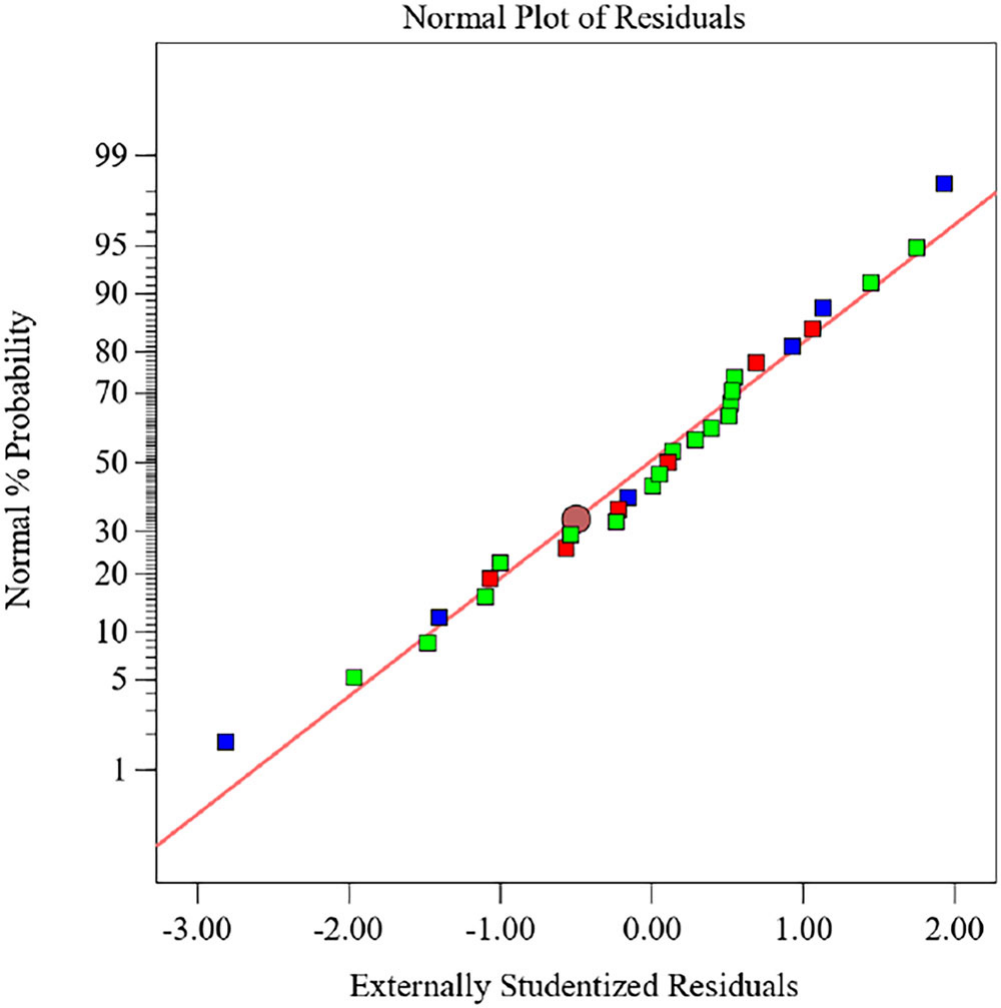
Residual model diagram.

Specifically, the DES mole ratio was found to fundamentally dictate the solvent viscosity and hydrogen‐bonding network. In isolation, an improper mole ratio could drastically increase viscosity, creating a severe mass transfer barrier. However, the highly significant interaction between the mole ratio and ultrasonic temperature (*p* < 0.01) indicates that the inherent mass transfer resistance can be effectively overcome. It is suggested that as the temperature is elevated, the reduced viscosity of the DES is coupled with intensified ultrasonic acoustic cavitation. Through this synergy, the viscous DES matrix is efficiently penetrated and disrupted by acoustic micro‐jetting, allowing anthocyanins to be liberated (Yuan et al. 2026).

Similarly, the significant interaction between the solid‐to‐liquid ratio and ultrasonic temperature (*p* < 0.01) demonstrates that sufficient interfacial turbulence, which is essential for consistent targeted desorption of BRA in varying solvent volumes, is maintained by acoustic cavitation. While the interaction between the solid‐to‐liquid ratio and water content (*p* < 0.05) plays a secondary role in modulating solvent polarity, the remaining nonsignificant interactions suggest that their individual effects dominate over any combined synergy. Ultimately, these comprehensive analyses confirm that maximal BRA recovery is not achieved by tweaking a single parameter but rather relies on the precise synergy between DES physicochemical properties and the mechanical forces of ultrasound, establishing a uniquely efficient extraction system.

#### 3.3.4 Optimization and Model Validation

Numerical optimization based on the established regression model predicted the optimal extraction conditions as follows: DES mole ratio of 1:2.06, solid‐to‐liquid ratio of 1:19.8 (g/mL), water content of 39.8%, and ultrasonic temperature of 50.8°C, with a predicted BRA yield of 37.47 mg/g.

Considering practical operation and instrumental limitations, the optimized conditions were adjusted to a DES mole ratio of 1:2, a solid‐to‐liquid ratio of 1:20 (g/mL), a water content of 40%, and an ultrasonic temperature of 51°C. Under these conditions, the experimentally obtained BRA yield was 37.44 ± 0.04 mg/g, which closely matched the predicted value. The small deviation between the predicted and experimental results confirms the high reliability and strong predictive capability of the response surface model.

### 3.4 In Vitro Antioxidant Activity of BRA

#### 3.4.1 Reducing Power Assay

**FIGURE 5:**
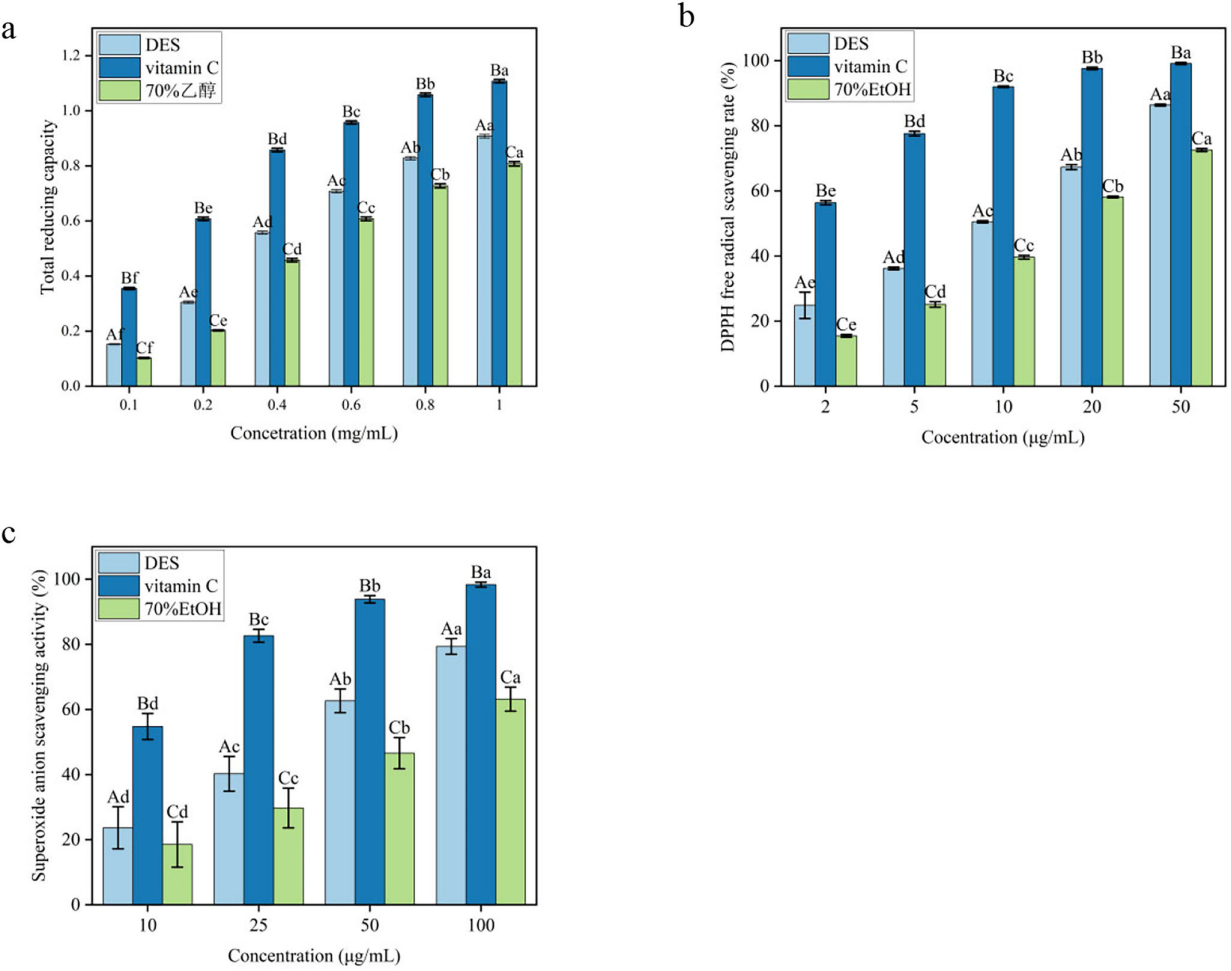
In vitro antioxidant activities of black rice anthocyanins: (a) reducing power, (b) DPPH radical scavenging activity, and (c) superoxide anion scavenging activity.

As illustrated in Figure 5a, the reducing power of DES‐extracted BRA, vitamin C (VC), and 70% ethanol extracts increased significantly with increasing concentration from 0.1 to 1.0 mg/mL (*p* < 0.05). The reducing power of DES–BRA increased from 0.153 ± 0.002 to 0.908 ± 0.007, while that of VC increased from 0.355 ± 0.004 to 1.108 ± 0.006. In contrast, the reducing power of the 70% ethanol extract ranged from 0.103 ± 0.002 to 0.808 ± 0.008. The corresponding IC_50_ values were 0.32 mg/mL for DES–BRA, 0.18 mg/mL for VC, and 0.45 mg/mL for the ethanol extract.

Although the reducing power of DES–BRA was lower than that of VC, it was markedly higher than that of the ethanol extract. This difference may be related to the distinct structural characteristics and redox behaviors of anthocyanins and ascorbic acid. In acidic DES systems, anthocyanins are expected to exist mainly in the flavylium cation form, which may influence their reducing performance. In contrast, the strong reducing activity of VC is generally attributed to its highly reactive 2,3‐enediol moiety, which facilitates electron donation (Sadowska‐Bartosz and Bartosz 2024).

#### 3.4.2 DPPH Radical Scavenging Activity

The DPPH radical scavenging activities of DES–BRA, VC, and the 70% ethanol extract are presented in Figure 5b. All samples exhibited concentration‐dependent scavenging activity in the range of 2–50 µg/mL (*p* < 0.05). At 50 µg/mL, DES–BRA achieved a scavenging rate of 86.38 ± 0.29%, compared with 99.14 ± 0.31% for VC and 72.57 ± 0.47% for the ethanol extract. The IC_50_ values were 22 µg/mL for DES–BRA, 1.8 µg/mL for VC, and 38 µg/mL for the ethanol extract.

These results indicate that DES–BRA possesses a strong DPPH (Sinopharm Chemical Reagent Co. Ltd.) radical scavenging ability, although it is less potent than VC. The antioxidant efficiency of anthocyanins is known to depend on the number and position of hydroxyl groups as well as glycosylation patterns, which may reduce the accessibility of phenolic hydroxyl groups and limit radical–molecule interactions.

#### 3.4.3 Superoxide Radical Scavenging Activity

As shown in Figure 5c, superoxide anion scavenging activity increased significantly with concentration (10–50 µg/mL) for all tested samples, including DES–BRA, VC, and the 70% ethanol extract (*p* < 0.05). At 50 µg/mL, the scavenging activity of DES–BRA reached 77.29 ± 2.42%, significantly outperforming the ethanol extract (60.12 ± 3.69%), although it remained inferior to VC (97.65 ± 0.77%).

The corresponding IC_50_ values were 55, 80, and 11 µg/mL for DES–BRA, the ethanol extract, and VC, respectively.

Collectively, these results indicate that DES–BRA exhibited markedly superior superoxide anion scavenging activity compared to the ethanol extract, suggesting that DES‐based extraction was more effective in preserving anthocyanins with antioxidant potential. This interpretation is consistent with recent studies showing that BRAs are important contributors to antioxidant activity, while DES/NADES systems can improve anthocyanin recovery, stability, and antioxidant performance relative to conventional organic solvents (Thakur et al. 2022).

### 3.5 In Vitro Hypoglycemic Activity

**FIGURE 6:**
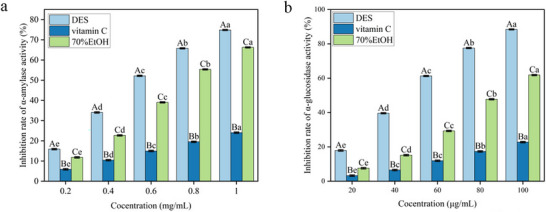
In vitro hypoglycemic activity of black rice anthocyanins: (a) α‐amylase inhibition and (b) α‐glucosidase inhibition.

α‐Amylase and α‐glucosidase are two key enzymes involved in carbohydrate hydrolysis and are therefore closely associated with postprandial glycemic regulation. As shown in Figure 6a, all tested samples inhibited α‐amylase activity in a concentration‐dependent manner over the range of 0.2–1.0 mg/mL, whereas DES–BRA exhibited the strongest inhibitory effect among the extracts. At 1.0 mg/mL, DES–BRA achieved an inhibition rate of 74.89 ± 0.29%, with an IC_50_ value of 0.60 mg/mL, which was lower than that of the ethanol extract (0.85 mg/mL) and VC (> 1.0 mg/mL).

A similar trend was observed for α‐glucosidase inhibition (Figure 6b). DES–BRA displayed a pronounced dose‐dependent effect in the range of 2–50 µg/mL, with the inhibition rate increasing from 17.39 ± 0.42% to 88.84 ± 0.33%. Its IC_50_ value (55 µg/mL) was markedly lower than those of the ethanol extract (80 µg/mL) and VC (> 100 µg/mL), indicating a substantially stronger inhibitory capacity. Taken together, these results suggest that DES‐based extraction outperforms 70% ethanol extraction in yielding anthocyanin‐rich fractions with enzyme‐inhibitory activity relevant to postprandial glucose control.

This interpretation is consistent with recent evidence showing that BRAs can contribute to glycemic regulation, at least in part, through the inhibition of carbohydrate‐digesting enzymes such as α‐amylase and α‐glucosidase, while the inhibitory potency of anthocyanin‐rich fractions is strongly influenced by anthocyanin structure and enzyme‐binding behavior (Sadowska‐Bartosz and Bartosz 2024). In addition, DES/NADES systems have been increasingly recognized as promising extraction media for anthocyanins because they can improve extraction efficiency and help preserve anthocyanin stability and bioactivity relative to conventional organic solvents (Thakur et al. 2022).

The pronounced enzyme inhibitory activities observed in the optimized UDE extracts can be mechanistically linked to their specific anthocyanin profile, as identified via LC‒MS analysis. C3G is the primary bioactive component responsible for α‐amylase and α‐glucosidase inhibition in black rice extracts (Leonarski et al. 2024). LC‒MS analysis revealed that the tailored DES–UAE microenvironment specifically enriched the C3G fraction. Consequently, the superior enzyme inhibitory capacity of the resulting extract can be directly attributed to the high relative abundance and preserved structural integrity of C3G achieved through this optimized process. Furthermore, it is postulated that the strong hydrogen‐bonding network of the DES solvent played a crucial protective role against the acoustic degradation of C3G during sonication (Dai et al. 2013), thereby ensuring that the target binding affinity of C3G to the active sites of digestive enzymes was effectively retained.

### 3.6 LC–MS Identification of Anthocyanins

**FIGURE 7:**
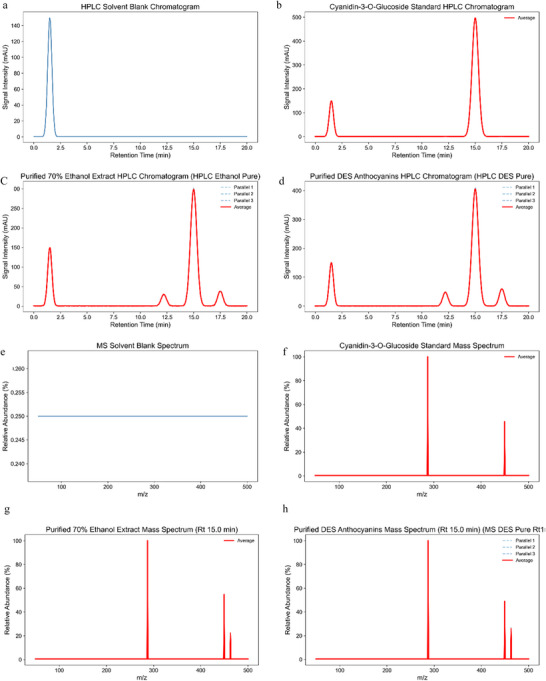
The total ion chromatogram of solvent (a), cyanidin‐3‐O‐glucoside standard (b), alcohol extract (c), and DES extract (d), fragment ion peak of solvent (e), fragment ion peak of cyanidin‐3‐O‐glucoside standard (f), fragment ion peak of alcohol extract (g), fragment ion peak of DES extract (h).

The anthocyanin profiles of DES and 70% ethanol extracts were analyzed by LC–ESI–MS. As shown in Figure 7, both extracts exhibited prominent [M+H]^+^ precursor ions in positive ion mode. The dominant fragment ions at *m*/*z* 287.1 and 301.1 correspond to the aglycone fragments of cyanidin, indicating that C3G (Shifeng Biotechnology Co., Ltd., Shanghai, China) is the major anthocyanin present in black rice extracts (Y. Wang et al. 2020).

Compared with the ethanol extract, the UDE showed a higher relative abundance of anthocyanin‐related ions, suggesting that the UDE more effectively enriched anthocyanins. The qualitatively similar fragmentation profiles between the two extracts provide preliminary evidence that DESs can serve as effective and selective solvents for anthocyanin enrichment without gross chemical alteration.

## 4 Conclusions

In this study, it is demonstrated that the integration of DESs with UAE provides a robust and sustainable strategy for recovering anthocyanins from black rice, which is driven by a critical physicochemical synergy. While the inherent high viscosity of DES typically acts as a mass transfer barrier in conventional extractions, this drawback can be effectively circumvented when it is coupled with UAE. The highly viscous DES matrix is effectively disrupted by the intense acoustic cavitation generated by ultrasound, whereby mass transfer resistance is significantly overcome, and the rapid targeted desorption of anthocyanins from the plant matrix is facilitated. When optimized by RSM, significantly higher extraction efficiencies were achieved by this synergistic ChCl‐lactic acid DES–UAE system compared to conventional ethanol‐based methods. Furthermore, through the subsequent integration of resin purification and LC–MS analysis, the high selectivity of this approach was confirmed; specifically, C3G‐rich fractions were enriched, while their chemical integrity was preserved. This structural preservation was shown to directly result in pronounced antioxidant and enzyme inhibitory activities. Compared to traditional organic solvent systems or standalone extraction technologies, a superior alternative is offered by this DES–UAE synergy, whereby extraction kinetics are simultaneously enhanced, and green chemistry principles are adhered to. Ultimately, a mechanistically sound and functionally relevant framework for developing black rice‐derived ingredients is provided by this work, thereby advancing green extraction technologies for cereal anthocyanins.

## Author Contributions

**Cuiqin Li**: writing – original draft, writing – review and editing, funding acquisition. **Yabin Shi**: funding acquisition, writing – review and editing. **Ziming Cao**: visualization. **Fengliang Liu**: writing – review and editing, supervision.

## Conflicts of Interest

The authors declare no conflicts of interest.

## Supporting information




**Supplementary Table**: jfds71120‐sup‐0001‐TableS1.docx

## Data Availability

The data that have been used are confidential.
