# Supplementary material for: Ultrasound‐Assisted DES Extraction of Black Rice Anthocyanins: Optimization, Characterization, and Bioactivity
Source: J Food Sci. 2026 May 8;91:e71120. doi: 10.1111/1750-3841.71120 (PMC13156430; doi:10.1111/1750-3841.71120)
Supplement: Supplementary file 1 — Supplementary Table: jfds71120‐sup‐0001‐TableS1.docx [file JFDS-91-0-s001.docx]

Factor levels and results of Box-Behnken experiments

| Std | Factor1 *A*:Mole ratio | Factor2 *B*:Solid-liquid ratio | Factor3 *C*:Water content | Factor4 *D*:Ultrasonic temperature | Response1 Anthocyanins yield(mg/g) |
| --- | --- | --- | --- | --- | --- |
| 1 | 1 | 15 | 40 | 50 | 29.73±0.89^a^ |
| 2 | 3 | 15 | 40 | 50 | 29.87±1.04 |
| 3 | 1 | 25 | 40 | 50 | 28.15±1.13 |
| 4 | 3 | 25 | 40 | 50 | 30.23±1.06 |
| 5 | 2 | 20 | 30 | 40 | 30.03±0.96 |
| 6 | 2 | 20 | 50 | 40 | 29.49±1.03 |
| 7 | 2 | 20 | 30 | 60 | 30.69±1.08 |
| 8 | 2 | 20 | 50 | 60 | 30.48±0.92 |
| 9 | 1 | 20 | 40 | 40 | 29.92±0.90 |
| 10 | 3 | 20 | 40 | 40 | 29.64±1.04 |
| 11 | 1 | 20 | 40 | 60 | 29.45±1.18 |
| 12 | 3 | 20 | 40 | 60 | 31.42±1.10 |
| 13 | 2 | 15 | 30 | 50 | 30.21±1.21 |
| 14 | 2 | 25 | 30 | 50 | 29.21±1.02 |
| 15 | 2 | 15 | 50 | 50 | 29.42±1.18 |
| 16 | 2 | 25 | 50 | 50 | 29.47±1.03 |
| 17 | 1 | 20 | 30 | 50 | 27.73±1.11 |
| 18 | 3 | 20 | 30 | 50 | 30.24±0.96 |
| 19 | 1 | 20 | 50 | 50 | 29.32±0.88 |
| 20 | 3 | 20 | 50 | 50 | 28.51±1.14 |
| 21 | 2 | 15 | 40 | 40 | 29.39±1.03 |
| 22 | 2 | 25 | 40 | 40 | 30.53±1.07 |
| 23 | 2 | 15 | 40 | 60 | 32.05±1.28 |
| 24 | 2 | 25 | 40 | 60 | 30.13±1.20 |
| 25 | 2 | 20 | 40 | 50 | 37.44±1.31 |
| 26 | 2 | 20 | 40 | 50 | 37.22±1.30 |
| 27 | 2 | 20 | 40 | 50 | 37.64±1.32 |
| 28 | 2 | 20 | 40 | 50 | 37.51±1.50 |
| 29 | 2 | 20 | 40 | 50 | 37.35±1.34 |

*Note*: ^a^ Means Mean ± SD, *n*=3.
